# Supplementary material for: Impact of Urea Addition and Rhizobium Inoculation on Plant Resistance in Metal Contaminated Soil
Source: Int J Environ Res Public Health. 2019 Jun 1;16(11):1955. doi: 10.3390/ijerph16111955 (PMC6603927; doi:10.3390/ijerph16111955)
Supplement: Supplementary file 1 [file ijerph-16-01955-s001.pdf]

Supplementary material

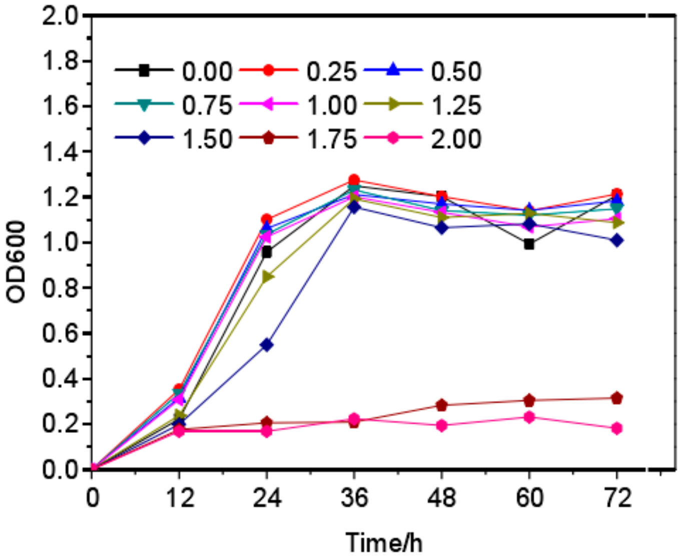

Figure S1. Growth curves of the rhizobium S. meliloti CCNWSX0020 under different Cu concentrations (mol L^{-1}).
